# Low-level laser therapy and anesthetic infiltration for orofacial pain in patients with fibromyalgia: a randomized clinical trial

**DOI:** 10.4317/medoral.21965

**Published:** 2017-12-24

**Authors:** Rebeca-Cecília-Vieira de Souza, Emerson-Tavares de Sousa, Kelly-Guedes-de Oliveira Scudine, Ussânio-Mororó Meira, Emanuel-Dias de Oliveira e Silva, Ana-Claudia-Amorim Gomes, Francisco-de Assis Limeira-Junior

**Affiliations:** 1 M. Sc. Department of Oral Surgery, Dental School, Pernambuco University, Recife, Pernambuco - Brazil; 2 M. Sc. Department of Pediatric Dentistry, Piracicaba Dental School, Campinas University, Piracicaba, Sao Paulo - Brazil; 3 M.D. Department of Medical Clinic, Hospital of Federal University of Paraiba, Joao Pessoa, Paraíba - Brazil; 4 Ph.D. Department of Oral Surgery, Dental School, Pernambuco University, Recife, Pernambuco - Brazil; 5 Ph.D. Department of Morphology, Dental School, Federal University of Paraiba, Joao Pessoa, Paraíba - Brazil

## Abstract

**Background:**

To compare the analgesic effect of anesthetic infiltration of lidocaine 2% and low-level laser therapy (LLLT) by GaAlAs into tender points of patients with orofacial pain and fibromyalgia (FM).

**Material and Methods:**

A randomized clinical trial was performed with adults (N=66) that were allocated into two groups (1:1): Group A received LLLT irradiation by Diode Laser GaAlAs (780nm) with expositions twice a week during six weeks and Group B was treated with anesthetic infiltration of lidocaine 2% without vasoconstrictor once a week for four weeks. The pain assessment included the Visual Analogic Scale (VAS) and tenderness to palpation.

**Results:**

No dropout and adverse effect was observed during the study. The pain decreased significantly in each group after the treatment (*p*=0.0001, β=1.0), even though no statistical difference was found between both treatments (*p*=0.46, β= 0.82). The presence of tender points decreased after both treatments, with responsively in some types of masticatory muscles (*p*<0.05) except posterior temporalis muscle. The patients’ perception showed that both treatments were effective and a few patients reported that the treatment did not improve welfare.

**Conclusions:**

The LLLT by GaAlAs and anesthetic infiltration of lidocaine 2% were equally effective to control orofacial pain in FM individuals.

** Key words:**Facial pain, myalgia, rheumatic disease, local anesthesia, phototherapy.

## Introduction

Fibromyalgia (FM) is a chronic musculoskeletal condition characterized by generalized pain with sensitivity to palpation in 11 or more of the 18 points at specific body locations (tender points) during at least 3 months (American College of Rheumatology - ACR) (1). Most patients experience fatigue accompanied by depression, anxiety, cognitive, sleep and mood disorders (2,3). Despite the fact that 0.5% to 5% of the entire population is diagnosed with the FM (4) and the significant relevance of this condition in the quality of life (5), the therapeutic approach remains in many cases insufficiently effective.

Although the etiology and pathophysiology of fibromyalgia are unclear, some hypotheses have been postulated. Some authors have described the etiology as a dysfunction of central nervous system, a reduction of activity of hypothalamic-pituitary-adrenal axis or a dysfunction of skeletal muscle nociception (6). Moreover, the incomplete understanding of the etiopathogenetic process of FM makes difficult to clinicians the decision-making, which should be associated with the treatment need of this disorder, the patient’s expectations, the health-related quality of life and the symptoms of patients.

As the cure for fibromyalgia is unknown, therapeutics should aim to reduce pain and provide a holistic approach for the patient and their family. In this context, a variety of treatment options to relieve fibromyalgia symptoms was proposed, such as instruction for the patient, psychotherapy, exercises, alternative medicine, pharmacologic therapy and LLLT (7,8). However, the unclear consensus on the optimal management choice and the low evidence of therapeutical protocols might be considered a challenge for the clinicians.

Recent studies suggest that the LLLT have efficient results of pain relief in patients with fibromyalgia (9,10-14). Among these research, only the study of Molina-Torres *et al.* (14) evaluated the effect of LLLT on the treatment of orofacial pain in individuals with FM, however, the effect size was moderate. Thus, there is no sufficient evidence that this type of treatment for orofacial pain in FM patients. On the other hand, the anesthesia injection might reduce pain originated from a concomitant tender point in selected patients with fibromyalgia who also experience myofascial pain (15). In fact, the anesthetic infiltration is the most commonly used treatment for myofascial pain syndrome characterized by the presence of trigger points (16). Even though there is no randomized clinical trial with sufficient evidence to support the use of this therapy in the management of tender points in the orofacial region of FM patients.

In this context, this study was conducted to investigate the analgesic effects of anesthetic infiltration of lidocaine 2% and LLLT by GaAlAs into tender points in patients with both orofacial pain and FM, associating these approaches with the patients’ perception of effectivity of treatment and well-being.

## Material and Methods

The present study was registered at the Brazilian clinical trial registrations (RBR-8k5yzn) and follows the CONSORT (Consolidated Standards of Reporting Trials) statement for randomized clinical trials (17).

- Ethical Considerations

This study was approved by Ethics Committee in Research of Federal University of Paraiba (CAAE: 08523712.6.0000.5183) and followed all the recommendations of Helsinki Declaration. The clinical approaches that might cause any possible discomfort or risk were fully explained to the individuals. All the individuals who accepted to participate in this study signed a free and informed consent.

- Sample 

**Figure 1:**
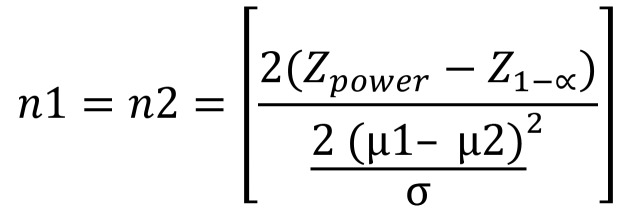
Formula.

The sample size calculation was carried out to provide a statistically significant difference between the experimental groups, using the two-tailed unpaired t-test. The standard deviations were based on the study of Demirkol *et al.* (18) and were considered the same for the two groups (σ1 = σ2= σ). The mean difference between the two populations was taken as µ1– µ2, estimating that the clinical relevance of 2 points of the Visual Analogue Pain Scale (VAS). The power was given as 0.9 and the level of significance was 0.05. The sample estimative was 56 individuals (n = n1 + n2), equally divided into two groups (n1 = n2), considering the application of the following formula as Röhrig *et al.*, (19) suggest: (Fig. 1).

The initial sample size calculation was increased by 20% to compensate possible losses during the treatment application, resulting in 66 volunteers necessary to perform such evaluation. Individuals with FM diagnosis and pain in the orofacial region were initially selected from a database of two centers of orofacial pain (one public and one private), located in a small capital of the Northeast of Brazil.

The sample was allocated into 2 groups (1:1): Group A (n=33) received LLLT and Group B (n=33) was treated by anesthetic infiltration. The block randomization was performed in Microsoft® Excel® 2016 software after the codification of each volunteer. This phase was accomplished by a research blinded and every member of the population had an equal chance of being chosen.

- Eligibility Criteria 

To be eligible for this study the patients had to fulfill the fibromyalgia diagnosis criteria from the American College of Rheumatology 1 and the myalgia diagnostic criteria for temporomandibular disorders (DC/TMD) (20). The diagnostic of fibromyalgia and orofacial pain were accomplished by a neurologist expert in chronic pain and a dentist, respectively. Additionally, the volunteers should have sufficient cognitive levels to understand procedures, follow instructions without the assistance of another person.

The exclusion criteria considered patients who changed their systemic medications 3 months before the beginning of the treatments, those who related the previous experience of an allergic reaction to lidocaine or do not agree to participate voluntarily in this research. During the treatments, if any alteration of the systemic medication were indicated by the responsible rheumatologist, the subject would be excluded from this research.

- Study Design and Interventions 

A parallel, controlled and randomized clinical trial design was performed with 66 adults of both genders (62 women and 4 men). The first author performed both types of treatment. Two evaluations, one qualitative e another quantitative, were carried out to test treatments’ effectiveness. The quantitative information was the measurement of orofacial pain intensity using the VAS. The VAS was self-completed by the respondent that placed a line perpendicular to the VAS at the point that represented their pain intensity. The pain scores reported by the patients ranged from 0 (no pain) to 10 (intense pain). These analyses were conducted on two occasions: at the beginning (1 day before the start) and at the end of the treatments (1 day after the ending). The measure of VAS considered the pain at rest or during the function in the last 15 days.

The qualitative information was the tenderness in the facial muscles as the primary clinical and diagnostic of local pain. Tender points were evaluated by an experient dentist (expert in Oral and Maxillofacial Surgery) that used manual palpation with the perpendicular pressure of two or three fingers on the surface of the skin, at approximately 4.0 kg/cm2 as suggested by Wolfe *et al.* (1). This evaluation was performed with the patient in supine position, following the assessment of bilaterally points: C posterior masseter muscle, [2] anterior masseter muscle, [3] anterior temporal muscle, [4] medium temporal muscle, [5] posterior temporal muscle. The presence and location of tender points were marked in a diagram that provided a specific condition of the patient status before the treatment. This information was used as a reference of the applications during all the treatment and was repeated at the end of the study.

Before the treatments, the skin of the individuals was disinfected with 70% alcohol and marked with a permanent marker at the points assessed in baseline evaluation. Treatment in Group A consisted of irradiation with Diode Laser GaAlAs (Twin Flex® MMOptics) based on a wavelength of 780nm, 50mW of power, Energy 2J (Dose 50J / cm2), spot 0,04cm2. The LLLT was applied precisely and continuously into the selected points during approximately 40 seconds. The patients were exposed to the laser application in a spot-skin distance of 1 cm while seated in a dental chair with their necks supported, two weekly sessions, during six weeks, for a total of twelve sessions. In the Group B, the individuals were treated by anesthetic infiltration of lidocaine 2% without vasoconstrictor (Lidostesim® SV). Carpule syringes with reflux and 30-G short needles (Unoject ®, DFL) were used in the procedure. A volume of 0.5 ml of lidocaine was infiltrated into each tender point and stretching was made after all the injections in order to help to distribute the solution across the muscle. The treatment in Group B was repeated once a week for four weeks.

The volunteers fulfilled a questionary after treatment conclusion to assess information regarding the perception of the effectiveness of both treatments and well-being. The questionnaire was structured with categorized questions (Yes or No) and following this sequence [1] In your opinion, do you think this treatment effective? [2] Do you think that this treatment improves your well-being? The questions were answered individually at the clinical office without any patient identification to control any possible bias.

- Statistical Analysis 

The primary outcome were the level of pain (VAS) and the incidence of pain before and after the treatment. The secondary outcome considered the individuals’ perception. The statistical analysis was carried out in SPSS software version 23.0 (SPSS, Inc., Chicago, IL, USA). Data were analyzed using descriptive and inferential statistics. Normality was obtained by the Shapiro-Wilk test. A comparative exploration of the treatments considering VAS was performed by Student t-test. The paired t-test was used to compare each treatment within the same group (before and after). The posthoc analysis accessed the β-1 value (effect size) using the software G*Power 3.1. McNemar’s test was used to access the difference between the presence of tenderness before and after the treatments. The level of significance adopted was 0.05 considering a two-tailed test.

## Results

All the volunteers completed the study. There were no adverse effects reported under the conditions of this study. The average age was 46.14 (± 10.91) years, with 76% of these individuals diagnosed with FM in the last 5 years. There was no significant difference between the age and gender of participants in both groups, with *p*-value > 0.05.  Table 1 shows a significant decrease of pain level in each group after the treatment (*p*=0.0001, β=1.0), even though, no statistical difference was found between both treatments (*p*=0.46, β= 0.82).

**Table 1:**
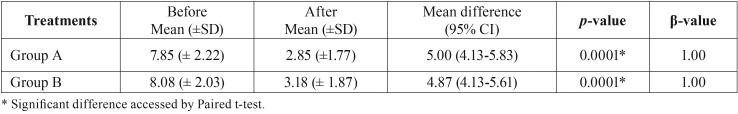
Average VAS for two study groups before and after treatment.

 Table 2 and  Table 3 demonstrates that there was a lower frequency of tender points after both treatments, with responsively in all types of muscles analyzed (*p*<0.05), except for the posterior temporal muscle that did not show significant difference after the applications (*p*>0.05).

**Table 2:**
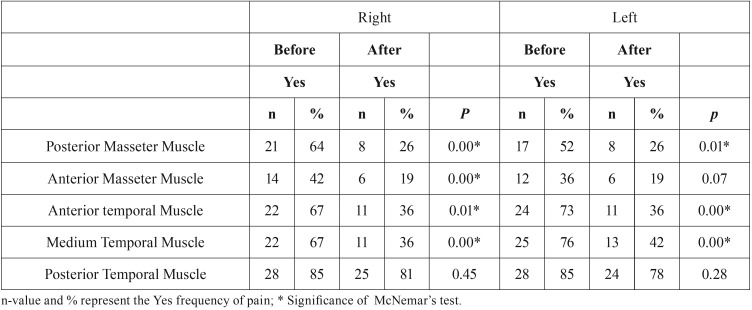
Frequency distribution of pain before and after treatment with LLLT (Group A) – Multiple responses.

**Table 3:**
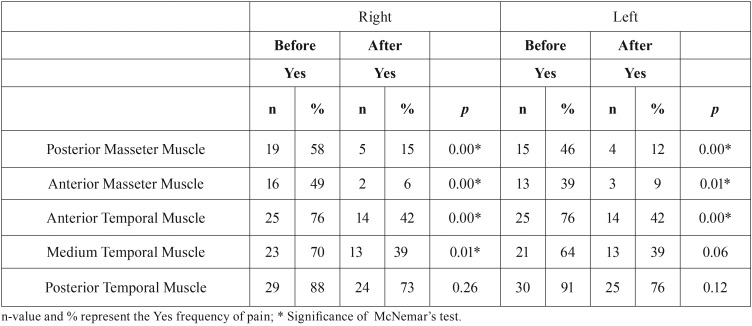
Frequency distribution of pain before and after treatment with Anesthetic Infiltration (Group B) – Multiple responses.

The patient’s perception showed that both treatments were effective, with 100% of positive response in Group A against 97% in Group B. Regarding the well-being after the treatment, 3% (n=1) of the patients in Group A related that the treatment did not improve welfare. This fact was also observed in Group B with higher proportion 18% (n=6) ( Table 4). None of the volunteers complained about the increase of pain at the conclusion of the study.

**Table 4:**
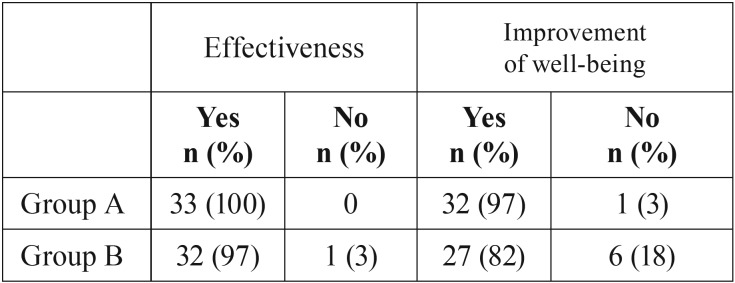
Patient’s perception of effectiveness and well-being provided by the treatments.

## Discussion

This randomized clinical trial was designed to specifically compare the treatment outcome of a pharmacological and a non-pharmacological approach. Two important parameters of orofacial pain were assessed as the intensity of pain by VAS and the presence and localization of tender points by palpation, both recommended by American Dental Association (ADA). Tender points are considered the primary clinical and diagnosis parameter of symptomatology of FM, which provides a reliable indicator of orofacial pain (21,22). In addition, VAS is a unidimensional, simple e frequently used method for the determination of pain intensity. The test-retest reliability of VAS has been shown to be good, as well the sensitivity to detect changes in pain after weeks with analgesic therapy (23).

Comparatively, in both groups, a significant decrease of pain level was observed (*p*<0.05), with 64% in Group A and 61% in Group B, without statistical difference between both treatments (*p*=0.46, β= 0.82). The LLLT (12-14) and anesthetic infiltration (24-26) has been studied in some modalities of therapies to pain remission in individuals with FM, although the comparison by controlled clinical trials and the superiority of each therapy are scarce, as well the relation of FM and orofacial pain. The only study that evaluated the LLLT in individuals with FM and orofacial pain 14 obtained a reduction of 44% of pain after the treatment using a protocol of 1 session per week during 12 weeks. Our study obtained better results with 2 sessions per week during 6 weeks.

While the mechanisms have not been completely explained, our study shows that LLLT and anesthetic infiltration clearly have an analgesic effect. In trigger points, the anesthetic infiltration modulates the pain by a reversible blocker of nerve conduction, a mechanical disruption of muscle fibers, an interruption of the positive feedback of chronic pain, a dilution and a clearance of nociceptive molecules by vasodilatory effect and volume administrated (27). On the other hand, the muscular photostimulation by LLLT modulates pain by peripheral nerve stimulation, microcirculation regulation, and release of anti-inflammatory agents (6,8).

Interestingly, these results demonstrate the relevance of orofacial pain in individuals with FM. Pain in the craniofacial area has not usually been considered in the diagnosis of FM, although, some investigations associate the facial pain with generalized muscle pain (28,29). In this study, there was a regular presence of tenderness in soft tissues, principally in temporal and masseter muscles. The study of da Silva (30) also observed a high prevalence of tender points in masseter and temporal muscle in fibromyalgia patients. The presence of FM and myofascial pain in this study emphasizes the importance of an individual assessment to identify and qualify comorbidities associated with FM, which will help the clinicians in disease control.

The considerable reduction of tender points in both groups was observed, although there are some of them that were not statistically responsive to the treatments. The anesthetic infiltration of lidocaine 2% and LLLT by GaAlAs showed to be ineffective to decrease the tenderness in posterior temporal muscle. This muscle is described as the most associated with facial pain in FM patients, with high patients’ complaint (26), what might be associated with the continuous activity function of temporalis muscle during the mandibular rest and elevation (31). Thus, the study of these treatments associated with the physiological function might be interesting to understand the muscle responsiveness in patients with FM.

The injections of lidocaine in patients with FM demonstrates a significant reduction of the facial pain intensity (24,26,32). However, the exact effect of anesthetic infiltration is difficult to estimate since the saline solution and dry needling is also considered a treatment (32). The LLLT is a physical modality of treatment that acts without the necessity of injection and administration of any pharmacological product. This fact is more important when the individual’s perception was considered (33) as it was observed in our study, which demonstrates that the patient’s perception is more favorable to the LLLT with 97% of improvement of well-being against 82% to anesthetic infiltration. However, the subjective implications of patients’ perception provide a bias of satisfaction, principally when considered the chronic pain suffering. In this context, the present study showed a tendency to the usage of LLLT being more accepted, non-invasive and promoting less discomfort and adverse effects.

This study has some limitations that emerged from difficulties naturally observed in clinical trials. Firstly, the absence of an extensive follow-up to investigate the durability of the analgesic effects of the applications. Secondly, the subjectivity of the parameters of clinical assessments represents the patients’ personal responses, which makes it difficult to standardize, considering that pain threshold is variable and unspecific. Thirdly, a control group was not included because the ethical impossibility of allocating individuals with high levels of pain in placebo group. Although it is important emphasizing that the control of the eligibility criteria and the follow-up promotes a more homogeneous sample that attenuates the influence of external factors (factors other than the treatment).

## Conclusions

It can be concluded that this particular type of LLLT using GaAlAs (780 nm, 50j/cm2, 50mW of power) with the protocol of applications twice a week during 6 weeks and the anesthetic infiltration of lidocaine 2% with once a week usage during four weeks were equally effective approaches to pain reduction in individuals with orofacial pain and FM. However, the usage of LLLT might be more interesting considering the patient’s comfort.
